# An Improved Method of Making Crowns

**Published:** 1904-02-15

**Authors:** Herbert Ziele


### AN IMPROVED METHOD OF MAKING CROWNS.

BY HERBERT ZIELE.

Prepare the root in the usual manner, so that its sides are parallel to a depth of one-sixteenth of an inch under the free margin of the gum. With a dentimeter and a fine steel wire procure the exact measurements of the root at the neck.

Take a piece of 22 karat gold, gauge 30, about one-thirty-second of an inch longer than such measurement, and cut to the depth of the crown required. Bevel the ends of this strip on opposite sides so that when annealed and bent in order to form a band the beveled edges will overlap and thus take up the one-thirty-second of an inch excess over exact measurement. Apply a minute quantity of borax (or preferably Sorosis soldering fluid), to this joint. As borax aids the fusing of gold it should be applied only to that part which requires fusing.

Heat may be directed to this joint either with a blowpipe or Bunsen burner until the two edges of the band are thoroughly fused together. This is what is known as "sweating," or autogenous soldering. The result will be a seemless band. This method is simplicity itself, and should be adopted for all kinds of gold crowns, being just as simple as soldering, and infinitely superior in every way.

Adjust this band to the tooth, festooning it so that it will pass under the free margin of the gum about one-sixteenth of an inch equally on its entire circumference. Bend and polish the edge so as to leave no roughness to cause irritation and subsequent trouble.

Trim the band short enough to free the opposing teeth when occluding, and contour carefully.

Now soften some hard wax (preferably Parr's fluxed), and fill the open end of the crown while it is in position, instructing the patient to bite and allowing the wax to remain a few moments until cool. Then remove band and wax together, cooling thoroughly by immersing in a glass of cold water.

Taste can here be displayed to advantage in carving the cups artistically in the wax. Do not, however, as in the Melotte system, expose the free edge of the band, but make it flush with the surface of same.

When the carving is completed readjust to the tooth. While the wax is still cold and hard, procure a small piece of platinum foil 1000 fine (ordinary inlay platinum), and about three-quarters of an inch square. Place it directly between the wax cusp and opposing teeth, instructing the patient to bite slowly. With a small instrument burnish the platinum over the buccal surface, the teeth being in apposition during this manipulation. Remove the band, wax, and platinum together, and cool again in the water.

Holding the band and platinum between the thumb and index finger of the left hand, with the right hand burnish the platinum over the wax on the mesial, lingual, and distal surfaces.

With a piece of ordinary binding wire bind the free edges of the platinum foil to the band at a distance of about one-eighth of an inch from the lower end of same (*do not* bind round cusps as is done in the ordinary crown). In this way the platinum foil is securely held to the hand.

Remove all excess of platinum above this wire with a pair of gum scissors.

Hold the crown with soldering pliers and burn out the wax over a Bunsen burner. As Parr's wax, if used, contains a small percentage of borax, any residue left will not be harmful and will not require to be removed by boiling water. If other wax is used a little borax will be necessary.

Cut up a number of *small* pieces of gold the same karat as that used for the band, and place several pieces on the inside of the platinum cusps.

Holding the band in the soldering tweezers, apply heat from a fine-pointed Bunsen burner or blowpipe directly on the outer surface of the cusps, causing the gold to flow readily over the entire inner surface of the platinum, sufficient gold being added to give a thick coating. If, however, an *extra strong* crown is required the cusps can be filled up with 22 karat solder.

Unwind the steel wire, and with the engine, stones and disks remove the platinum, which, as it is only one-thousandth of an inch thick, will not affect the bite in any way.

The advantages claimed for this crown are many. It will not show signs of tarnishing as is seen in crowns having soldered joints; the bite will be more perfect than those with cusps made from a die plate, and as perfect as those made with Melotte's or similar systems requiring dies or counter dies; moreover, less time is required in its manufacture.—*Stomatologist.*

